# Histological analysis of sleep and circadian brain circuitry in cranial radiation-induced hypersomnolence (C-RIH) mouse model

**DOI:** 10.1038/s41598-022-15074-0

**Published:** 2022-07-01

**Authors:** Dorela D. Shuboni-Mulligan, Demarrius Young, Julianie De La Cruz Minyety, Nicole Briceno, Orieta Celiku, Amanda L. King, Jeeva Munasinghe, Herui Wang, Kendra A. Adegbesan, Mark R. Gilbert, DeeDee K. Smart, Terri S. Armstrong

**Affiliations:** 1 Neuro-Oncology Branch, National Cancer Institute, National Institutes of Health, Bethesda, MD USA; 2 Mouse Imaging Facility, National Institute of Neurological Disorder and Stroke, NIH, Bethesda, MD USA; 3 Radiation Oncology Branch, Center for Cancer Research, National Cancer Institute, National Institutes of Health, Bethesda, MD USA

**Keywords:** Cancer therapy, CNS cancer

## Abstract

Disrupted sleep, including daytime hypersomnolence, is a core symptom reported by primary brain tumor patients and often manifests after radiotherapy. The biological mechanisms driving the onset of sleep disturbances after cranial radiation remains unclear but may result from treatment-induced injury to neural circuits controlling sleep behavior, both circadian and homeostatic. Here, we develop a mouse model of cranial radiation-induced hypersomnolence which recapitulates the human experience. Additionally, we used the model to explore the impact of radiation on the brain. We demonstrated that the DNA damage response following radiation varies across the brain, with homeostatic sleep and cognitive regions expressing higher levels of γH2AX, a marker of DNA damage, than the circadian suprachiasmatic nucleus (SCN). These findings were supported by in vitro studies comparing radiation effects in SCN and cortical astrocytes. Moreover, in our mouse model, MRI identified structural effects in cognitive and homeostatic sleep regions two-months post-treatment. While the findings are preliminary, they suggest that homeostatic sleep and cognitive circuits are vulnerable to radiation and these findings may be relevant to optimizing treatment plans for patients.

## Introduction

Sleep disturbances (SD) are among the most common symptoms reported by primary brain tumor (PBT) patients^1,2^ and can impair quality of life and treatment tolerance. SD is defined as perceived or actual alterations in sleep that can result in impaired daytime function, difficulty falling and staying asleep, and daytime hypersomnolence^3,4^. An increased incidence of sleep disturbances is associated with oncologic therapies^5,6^, including cranial radiotherapy, which is often the standard of care for PBT patients^7–11^. Radiotherapy functions by directly damaging the DNA of tumor cells, triggering apoptosis^12,13,13^ or mitotic catastrophe, however, healthy brain tissue is also damaged during treatment^14,15^. Brain injury is common in patients receiving radiotherapy^15^ and can be classified into three phases: acute reactions (two weeks); early delayed reactions (two weeks to six months); and late delayed reactions (several months to years). Clinically, the Radiation Therapy Oncology Group (RTOG) grading scale distinguishes injury between acute (<3 months) and late toxicity (>3 months) after the completion of treatment^16^. Understanding how different regions of the brain are injured and the symptoms this damage can trigger will provide insight into improving treatment strategies.

The link between healthy brain injury caused by radiation and symptoms is best demonstrated in the cognitive literature^17–19^. In the PBT patient population, neurocognitive decline is correlated with the dose of radiation administered to the hippocampus^20^. The hippocampi have also been shown to shrink in size post-treatment^21^ and hippocampal-sparing techniques may improve the incidence of cognitive decline^22–24^. Despite this well-established impact of radiation damage on the hippocampus, to our knowledge, no studies have examined the impact of radiation damage in other non-cognitive brain regions including the brain sleep circuits. Sleep in the brain is regulated by two pathways: a homeostatic drive that functions via sleep pressure and a circadian drive that modulates the timing of sleep and arousal^25^. The master clock that controls circadian rhythms in mammals is found in the suprachiasmatic nuclei (SCN) of the anterior hypothalamus^26–28^. Our previous work has shown that circadian clock genes are a good predictor of the development of radiation-induced hypersomnolence in PBT patients^29^. Damage to the circadian and homeostatic sleep regions of the brain caused by radiation may, therefore, be important in the development of SD.

To test the hypothesis, we first developed a mouse model of cranial-radiation-induced hypersomnolence (C-RIH). Our model demonstrates that activity and sleep are affected by cranial irradiation in a manner that recapitulates the human experience in two experiments: (1) a short-term dose response curve across radiation intensities and (2) longer-term monitoring over one month. Histological and imaging analysis of our mice post-radiation also identifies the variable response to radiation across the mouse brain, a finding further supported by *in vitro* examination of cells from the SCN and cortex.

## Results

### Cranial irradiation impacts mouse behavior in a dose response manner

**Figure 1:**
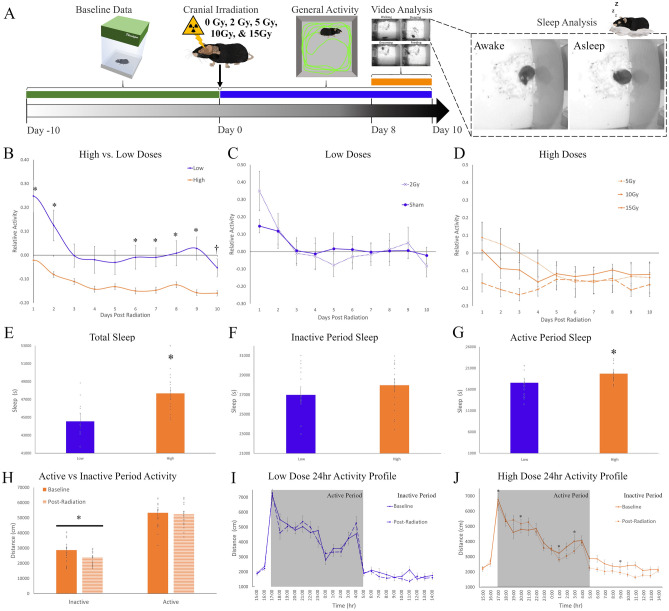
Radiation dose response analysis in mouse CRIH model. (**A**) The dose response curve experimental timeline with mice exposed to 0, 2, 5, 10 or 15 Gy radiation (n = 6/group). Mice were monitored in Noldus Phenotyper™ cages for 10 baseline days (green), general activity data (blue) were then collected for 10 days post-irradiation and other specific behaviors where characterized using the Mouse Behavioral Module from video from day 8–10 (orange). These behaviors include drinking, grooming, feeding and sleep-like behavior (inlet). (**B**) Relative general activity levels mapped across 10 days post-treatment when groups were divided into high (5, 10 & 15 Gy; orange) or low (0 & 2 Gy; blue) radiation levels. Activity levels were standardized to produce relative activity values by dividing the 24 h of post-treatment for all 10 days by the average baseline activity levels. Comparisions of high and low radiation levels had significant main effects (time: F(9,234) = 11.686, *p* < 0.001; radiation dose: F(9,234) = 7.703, *p* = 0.010) and an interaction between time and level of radiation (F(9,234) = 2.195, *p* = 0.023). Posthoc tests (tukey) were significant if *p* < 0.05 (**C**) Low dose groups remained close to baseline levels (0.0), except for the first two days post-treatment. (**D**) High dose groups across 10 days post-treatment showed least a 10% decrease in activity by the 6th day post-radiation, with the greatest effects observed in the 10 (dashed line) and 15 (solid line) Gy. **E** Total sleep levels were significantly higher in the high dose animals than the low dose mice in the last 3 days of monitoring (t(30) = 2.318, *p* = 0.027). (**F**) During the inactive period, daytime, mice did not significantly differ in levels of sleep between high and low doses (t(30) = 0.788, *p* = 0.437). (**G**) During the active period, nighttime, mice did have significantly more sleep in the high dose group when compared to the low dose group (t(30) = 2.900, *p* = 0.007). (**H**) Comparisons in activity level during the active vs inactive periods of 15 Gy at baseline and post-treatment with a significant interaction (F(1,17) = 7.328, *p* = 0.015). Mice in the high dose groups had significantly less activity during the inactive period (daytime). (**I**) Twenty-four profile of activity for the low dose group during the baseline and post-treatment were similar. Grey panels indicate the active period, while the white portion of the graph are the inactive period. There was a main effects of time (F(23,299) = 66.808, *p* < 0.001) but not a main effect of radiation (F(1,13) = 0.178, *p* = 0.680) or interaction between time and radiation (F(23,299) = 1.620, *p* = 0.160). j. Twenty-four profile of activity for the high dose group during the baseline and post-treatment were different, with post-radiation showing less activity. Statisitcs showed a significant interaction between radiation and time (F(23,391) = 3.028, *p* = 0.004), with a main effect of time (F(23,391) = 98.257, *p* < 0.001) but not for radiation (F(1,17) = 2.952, *p* = 0.104. Posthoc tests (tukey) were significant if *p* < 0.05. Significance was defined as *p* < 0.05 and indicated by *.

In the first set of experiments, we examined the impact of cranial irradiation on behavior. To determine the optimal dose required to induce C-RIH, we looked at the early effects of radiation (10 days; Fig. 1A) using a wide range of irradiation doses (Sham, 2, 5, 10, and 15 Gy) administered as a single treatment (Sham n=8; 2-15 Gy n = 6). We further assessed the effects on the animals stratified into high dose (5, 10, and 15Gy) and low dose (0 and 2 Gy) groups. The higher doses mirror those used by other investigators demonstrating equivalent therapeutic effect for doses 10 Gy and above in mice^30^. To account for pre-treatment inter-subject variability among the mice, post-radiation general activity over a 24 h period was standardized to the baseline levels of activity within each. General activity at baseline was not significantly different across the five dose groups (F(4,31) = 0.843, *p* = 0.510); this remained the case when grouping animals into high/low radiation groups (t(30) = 0.345, *p* = 0.733), with distance traveled averaging 74,447.00 ± 4615.38 and 72,318.26 ± 3707.38 cm for high and low dose groups, respectively. Assessing post-radiation effects on activity by high/low level of radiation and across time points, we found significant main effects (time: F(9,234) = 11.686, *p* < 0.001; radiation level: F(9,234) = 7.703, *p* = 0.010) and an interaction between time and level of radiation (F(9,234) = 2.195, *p* = 0.023 (Fig. 1B). Sham and 2 Gy radiation had an initial increase in activity, 15 ± 3% and 34±11% respectively, when returned to clean cages, an effect that is commonly observed in mice^31^ and which lasted two days. After this period, the activity levels of the low dose animals returned to baseline levels for the remaining 8 days (Fig. 1C). No initial activity increase was observed for the high dose animals, except for a small 8 ± 8% activity increase for the 5 Gy animals. Animals who received high doses of radiation had suppressed activity after the 5th day post-exposure (Fig. 1B, *p*<0.024). The average activity in the first two days across the 5 radiation dose groups was significantly different (one-way ANOVA, F(4,31) = 4.495, *p* = 0.007), with the 10 (*p* = 0.001) and 15 Gy (*p* = 0.019) groups having less activity than the sham and 2 Gy groups. For the remaining 8 days, all high radiation dose groups had at least a 10% decrease in activity by the 6th day post-radiation while the low doses groups remained around baseline. Within the higher dose groups, 10 and 15 Gy had an earlier drop of activity under baseline levels than the 5 Gy group (Fig. 1D). In summary, we observed reduced activity in a dose-dependent manner with therapeutic (high) doses associated with the greatest reductions.

To understand the specific changes in behavior that occur during the period of activity suppression, the last three days of video for each animal was processed using the Ethovision 14 Mouse Behavioral Module software. Our previous work^32^ has demonstrated that automatic scoring using the software was equivalent to manual scoring by an experienced sleep researcher for all behaviors (grooming, eating, drinking, and sleep-like behavior). Total sleep-like behavior was significantly different between the high and low dose groups (t(30) = 2.318, *p* = 0.027), with the high radiation groups sleeping more than the low dose groups (Fig. 1E). Patients suffering from C-RIH have heightened daytime sleep, i.e.**,** napping, during the time they are supposed to be active^1,2^. To determine if radiation impacted sleep during the inactive (day) or active (night) time in our mouse model, we separated the data by lighting condition. Mice are nocturnal and spend most of the time sleeping when lights are on during the day and are active during the nighttime^33–35^. The high dose group slept more during the inactive period, a 3% increase from the low dose (Fig 1F), but this difference was not significant (t(30) = 0.788, *p* = 0.437). Nighttime, on the other hand, had a larger increase (12.2%, Fig. 1G) of sleep in the high dose group and was significantly different than the low dose group (t(30) = 2.900, *p* = 0.007). Other behaviors, such as eating and drinking, did not show a significant difference between high and low dose animals. However, more grooming behavior tended to happen in the low dose group, especially in the active period with 22.3% more grooming than high dose (Supplementary Figure S1a–c) but the groups were not significantly different (t(30) = 1.607, *p* = 0.119). General activity was also compared during the active and inactive period (Fig. 1H), only the high dose group had a significant interaction between the active/inactive phase and the baseline/post-radiation activity levels (F(1,17) = 7.328, *p* = 0.015). Activity was plotted across 24 h for low (Fig. 1I) and high (Fig. 1J) radiation groups with lines representing the baseline and post-radiation profiles. The low radiation group had main effects of time (F(23,299) = 66.808, *p* < 0.001) but not a main effect of radiation (F(1,13) = 0.178, *p* = 0.680) or interaction between time and radiation (F(23,299) = 1.620, *p* = 0.160). The high radiation group had an interaction between radiation and time (F(23,391) = 3.028, *p* = 0.004), with a main effect of time (F(23,391) = 98.257, *p* < 0.001) but not for radiation (F(1,17) = 2.952, *p* = 0.104). Mice in this group had significant suppression of post-radiation activity during the inactive period (t(17) = 2.551, *p* = 0.021). Circadian parameters were measured using methods established in Shuboni-Mulligan et al.^32^; we plotted distance traveled in 10min intervals and quantified amplitude, activity onset, offset, and precision for the final 3 days (days 8–10) of analysis and compared it to the initial 3 baseline days. None of the circadian variables analyzed were statistically significant (Supplementary Table S1). Overall, radiation levels had differential effects and higher doses produced similar behavioral changes as observed in humans: i.e.**,** lower activity and more sleep during the active phase.

### Longer-term effects of irradiation are observed in sleep and general activity patterns

**Figure 2:**
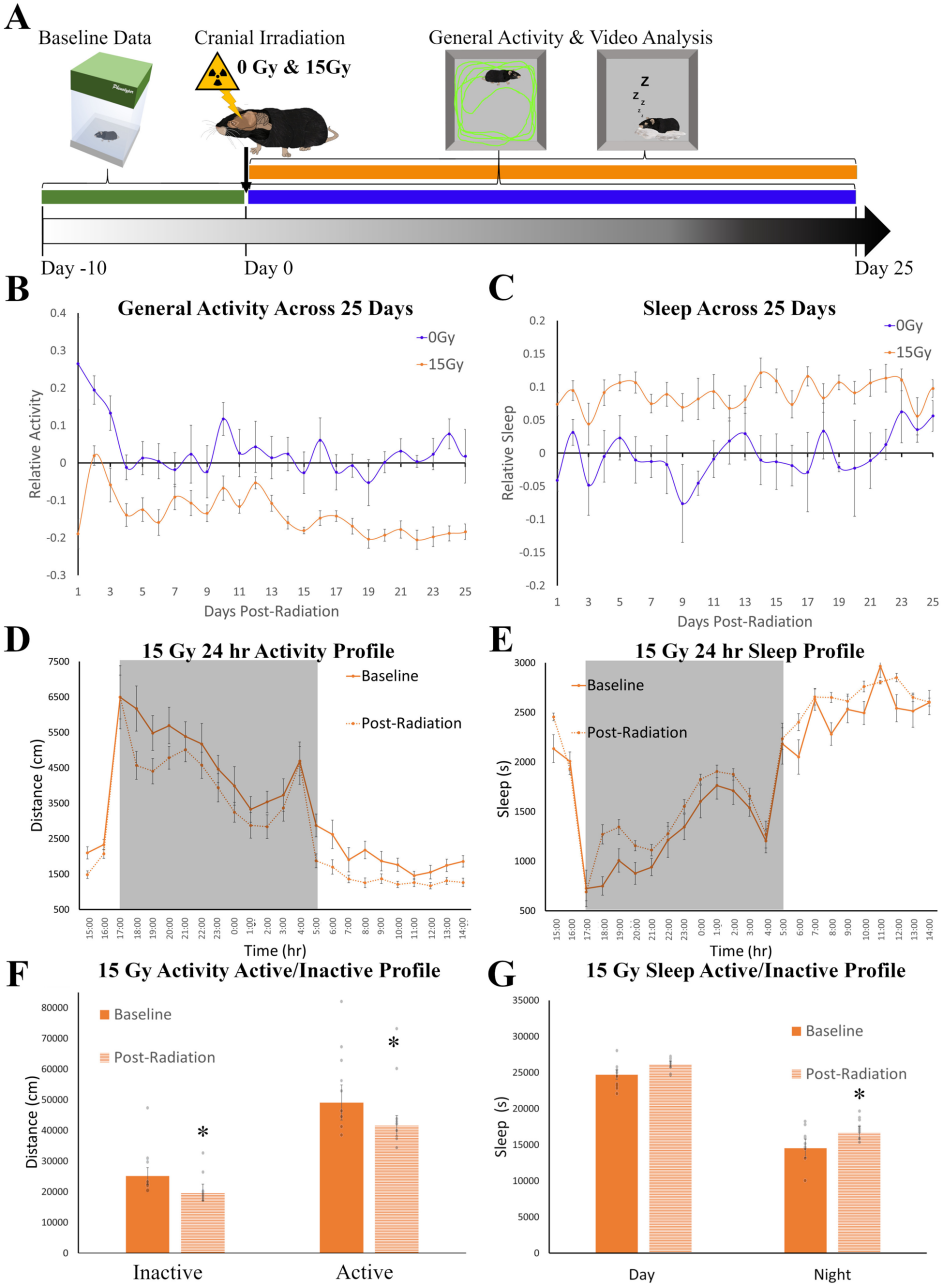
Long-term monitoring of sleep and activity across 25 days post-irradiation. (**A**) The long-term monitoring experimental timeline with mice exposed to 0 or 15 Gy radiation (n = 8/group). Mice were monitored in Noldus Phenotyper™ cages for 10 baseline days (green), then general activity data (blue) and video sleep analysis (orange) were then collected for 25 days post-irradiation. (**B**) Relative activity levels across 25 days when summed over 24 h. Mice exposed to 0 Gy (blue line) remained close to baseline levels (0.0) while the 15 Gy group (orange line) had decreased levels of activity with progressively more suppression across time. There was a significant main effect of radiation (F(1,2) = 26.840, *p* = 0.035) and no significant effect of time (F(24,48) = 0.998, p = 0.445) or interaction between time and radiation (F(24,48) = 0.702, p = 0.547). (**C**) Relative sleep levels across 25 days when summed over 24 h. Mice exposed to 0 Gy (blue line) again remained close to baseline levels while those exposed to 15 Gy (orange line) had higher levels of sleep post treatment which was sustained over time. There was a main effect of radiation (F(1,2) = 100.358, *p* = 0.010) and no significant effect of time (F(24,48) = 1.109, *p* = 0.413) or interaction between time and radiation (F(24,48) = 0.884, *p* = 0.478). (**D**). Daily profile of raw activity across 24 h for the 15 Gy group for baseline (solid line) and post-treatment (dotted line). Grey panels indicate the active period during the 24 period, while the white portion of the graph are the inactive period. Mice are nocturnal and are active primarily during the dark phase, night. Post-treatment activity levels in 15 Gy mice were suppressed during both phases. (**E**) Daily profile of raw sleep across 24 h for the 15 Gy group for baseline (solid line) and post-treatment (dotted line). Mice sleep primarily during the light phase, daytime. Post-treatment sleep levels appear to be heightened primarily during the active phase of the 24 h period. (**F**) Comparisons in activity level during the active/inactive periods of 15 Gy at baseline and post-treatment. There were significant decreases in activity post-treatment for both the inactive and active periods with a main effects of time (Active/Inactive; F(1,8) = 90.781, *p* < 0.001) and treatment (Baseline/Post-Treatment; F(1,8) = 35.646, *p* < 0.001), but no interaction (F(1,8) = 0.543, *p* = 0.482). (**G**) Comparisons in sleep level during the active/inactive periods of 15 Gy at baseline and post-treatment. There were significant decreases in sleep post-treatment for only the active period with main effects of time (Active/Inactive; F(1,8) = 158.219, *p* < 0.001) and treatment (Baseline/Post-Treatment; F(1,8) = 29.629, *p* < 0.001), but no interaction (F(1,8) = 0.370, *p* = 0.560). Significance was defined as *p* < 0.05 and indicated by *.

To further explore the longer-term behavioral changes that occur post-treatment, we exposed a second cohort of mice to a high dose of radiation (15 Gy) and monitored behaviors for a longer period, 25 days (Fig. 2A). The baseline activity (t(13) = 0.257, *p* = 0.801) and sleep (t(13) = 1.359, *p* = 0.197) of sham mice (0 Gy, n = 6) were not significantly different from 15 Gy irradiated mice (n = 10). Relative sleep and activity were calculated for each mouse across the 25 days post-irradiation by dividing the total daily activity or sleep per day by the baseline levels (Fig. 2B, C). Again, as anticipated general activity showed a significant spike in the first two days post-irradiation for sham mice (t(10) = 2.965, *p* = 0.014) that was not observed in the 15 Gy irradiated animals. Post irradiation comparisons between the 15 Gy and sham mice across time had a significant main effect of radiation (F(1,2) = 26.840, *p* = 0.035) but no significant effect of time (F(24,48) = 0.998, *p* = 0.445) or interaction between time and radiation (F(24,48) = 0.702, *p* = 0.547). Overall, irradiated mice had a 14.77 ± 0.91% decrease in the amount of activity when compared to their baseline levels (Fig. 2B), while sham mice remained close to baseline (+1.99 ± 0.97%). Within the 15 Gy group, initial levels of activity suppression were like the first experiment with a ~10% decrease in activity but further decreased to ~20% after the second week. Sleep-like behavior also had a significant main effect by ANOVA of radiation (F(1,2) = 100.358, *p* = 0.010) and no significant effect of time (F(24,48) = 1.109, *p* = 0.413) or interaction between time and radiation (F(24,48) = 0.884, *p* = 0.478). In particular, mice in the irradiated group spent significantly more time sleeping than sham mice (Fig. 2C). Again, we mapped out the daily profiles of each behavior to understand how treatment altered timing and distribution parameters (Fig. 2D,E). Activity appeared to be suppressed in both the active and inactive phases, while sleep increases were mostly observed in the active period. In the Inactive/Active comparison, general activity in the 15 Gy mice (Fig. 2F) had significant main effects of time (Active/Inactive; F(1,8) = 90.781, *p* < 0.001) and treatment (Baseline/Post-Treatment; F(1,8) = 35.646, *p* < 0.001), but no interaction (F(1,8) = 0.543, *p* = 0.482). This indicated that both during the active and inactive period, activity was suppressed in the mice in a similar pattern. Sleep followed a similar Active/Inactive pattern as activity (Fig. 2G) with significant main effects of time (Active/Inactive; F(1,8) = 158.219, *p* < 0.001) and treatment (Baseline/Post-Treatment; F(1,8) = 29.629, *p* < 0.001), but no interaction (F(1,8) = 0.370, *p* = 0.560). However, there were greater increases in sleep after 15Gy radiation during the active period (+14.53%) when compared to the inactive period (+5.89%). Grooming was not significantly different in these experiments between the two groups (Supplementary Figure S2). Findings from the second behavioral experiment, therefore, mirror the results shown in the first experiment but demonstrate that the behavioral changes persist across the longer timeframe of 25 days.

### Mapping of acute DNA damage response within the brain post-irradiation identifies region-specific effects in cognitive and sleep brain regions

**Figure 3:**
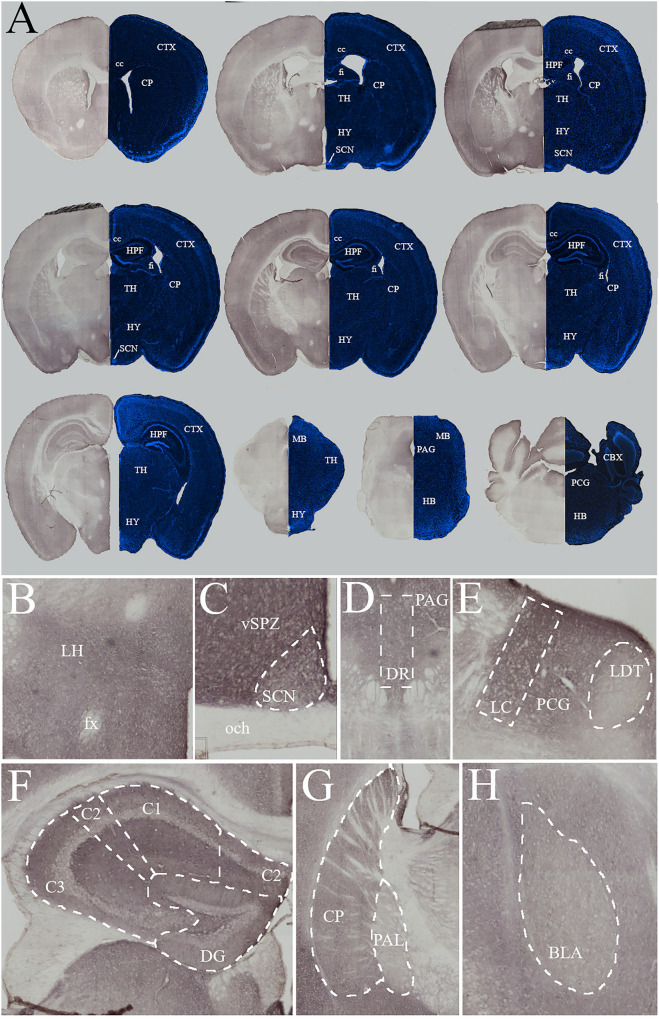
Histological Analysis of γH2AX across the whole brain 1 h post-radiation. (**A**) Full rostral to caudal mapping of γH2AX (Left) and DAPI (Right) in coronal sections. 1 h post-radiation (15 Gy, n = 4). Chromogen γH2AX staining indicates the levels of DNA damage and varies across different regions of the brain. Generally, high levels of staining are seen across most of the neocortex and the hypothalamus, while the thalamus, midbrain, and hindbrain have a patchwork of staining with many regions unstained and only a few areas with similar levels of γH2AX to the cortex. Fluorescent DAPI staining indicates the number of nuclei within the tissue, the number of nuclei do not apprear to drive the effects of γH2AX staining. Abbreviation: cc, corpus callosum; CTX, cortex; CP, caudoputamen; fi, fimbria;TH, thalamus; HY, hypothalamus; SCN, suprachiasmatic nucleus; HPF, hippocampal formation; MB, midbrain; HB, Hindbrain; PAG, periaqueductal gray; PCG, pontine cental grey; and CBX, cerebellum. (**B**) The hypothalamus has high levels of γH2AX staining, including a regions critical for homeostatic sleep the lateral hypothalamus (LH). (**C**) One region that had lower levels of staining in the hypothalamus was the SCN, the master clock that modulates the expression of circadian rhythms in mammals. Directly above the SCN is a region critical for transmitting the circadian rhythm signals from the SCN to the body, the vental subparaventricular zone (vSPZ). This regions shows high levels of staining when compared to the SCN and optic chiasm (och). (**D**) In the midbrain the PAG and adjacent dorsal raphe (DR), which is critical for the homeostatic sleep pathway, both showed similar high levels of staining. (**E**) Further caudal in the hindbrain, the PCG and adjacent locus ceruleus (LC), which is also critical for the homeostatic sleep pathway, both also showed more γH2AX staining than the laterodorsal tegmental nucleus (LDT). (**F**) The hippocamal formation showed some of the highest stainng levels across the brain. Similar levels appeared in all subregions of the hippocamus including C1-3 and the dentate gyrus (DG). (**G**) CP and Pallidum (PAL) show a difference in staining levels between two regions of the neocortex, with lower levels in the later. (**H**) Lower levels of staining was also observed in the basolateral amygdala (BLA), a region important for fear learning.

To characterize the initial radiation response of different regions of interest, brains were collected from animals, euthanized 1 h after exposure to 15 Gy (n = 4) or sham (0 Gy, n = 2) cranial irradiation. Whole brains were sectioned and processed against an antibody for γH2AX (Fig. 3A, left sections), a marker for DNA damage caused by ionized radiation^36,37^. A replicate set of brains sections were stained with DAPI to demonstrate the number of cell nuclei in regions of interest (Fig. 3A, right sections). The number of cell nuclei did not drive the level of γH2AX staining; regions with the highest number of cell nuclei, such as the SCN, did not have higher γH2AX levels. Similar qualitative descriptions of cell nuclei densities have been reported on the differences in cell numbers across the brain^38,39^. The level of cell density in the SCN of Rhesus monkeys^40^ was reported as 171,551 cells/mm^3^ as compared to the total hypothalamus of 131,459cells/mm^3^. Tissue from our animals that received sham irradiation was processed alongside the brains of irradiated mice as a sham and showed no background staining (Supplementary Figure S3), which indicates that the staining observed in the irradiated animals was specific to the antibody and radiation condition. As expected, the hippocampus was one of the regions that contained the heaviest γH2AX staining (Fig 3F).

When examining general staining across the brain, high levels of staining were seen across most of the neocortex and the hypothalamus. The lateral hypothalamus, which is home to orexinergic neurons important for triggering sleep, was included in the densely stained regions (Fig. 3B). One lone nucleus within the hypothalamus that appeared devoid of staining was the SCN (Fig. 3C, Supplementary Figure S4a), which is resilient to many other methods of chemical lesioning^41–44^. The thalamus had a patchwork of staining, with higher levels of γH2AX in rostral sections and the medial regions, specifically the paraventricular nucleus of the thalamus (PV, Supplementary Figure S5a). Very little staining was observed in the geniculate complex (Supplementary Figure S5b) of the thalamus, a region associated with vision and the circadian light response. The midbrain and hindbrain generally had sparse staining except for regions near the ventricular system, a pattern that has been suggested in the proton beam therapy literature^45^. Regions associated with the homeostatic sleep circuit in these areas had high levels of staining, including the dorsal raphe (Fig. 3D) and the locus coeruleus (Fig. 3E). Staining patterns in sleep regions appear to favor greater acute γH2AX response within the homeostatic system and not in areas related to circadian rhythms. Others have demonstrated that the region is sensitive to radiation and shown that neural stem cells in the dentate gyrus (DG) are particularly sensitive^17,46,47^. Our staining appears across the different regions of the hippocampus (Fig. 3F), including the CA1, CA2, CA3, CA4, and Dentate gyrus (DG). Other cognitive function-related brain regions also showed higher staining, including the cortex (CTX), cerebellum (CBX), and caudoputamen (CPu). The basolateral amygdala (BLA, Fig. 3H) and the globus pallidus (GPe, Fig. 3G), on the other hand, had less staining than the adjacent regions. We quantified the optical densities, which demonstrates the level of γH2AX staining, of these regions using ImageJ software across the brain (n = 4, Supplementary Figure S4b).

### Differential regional radiation response is observed in vitro for astrocytes

**Figure 4:**
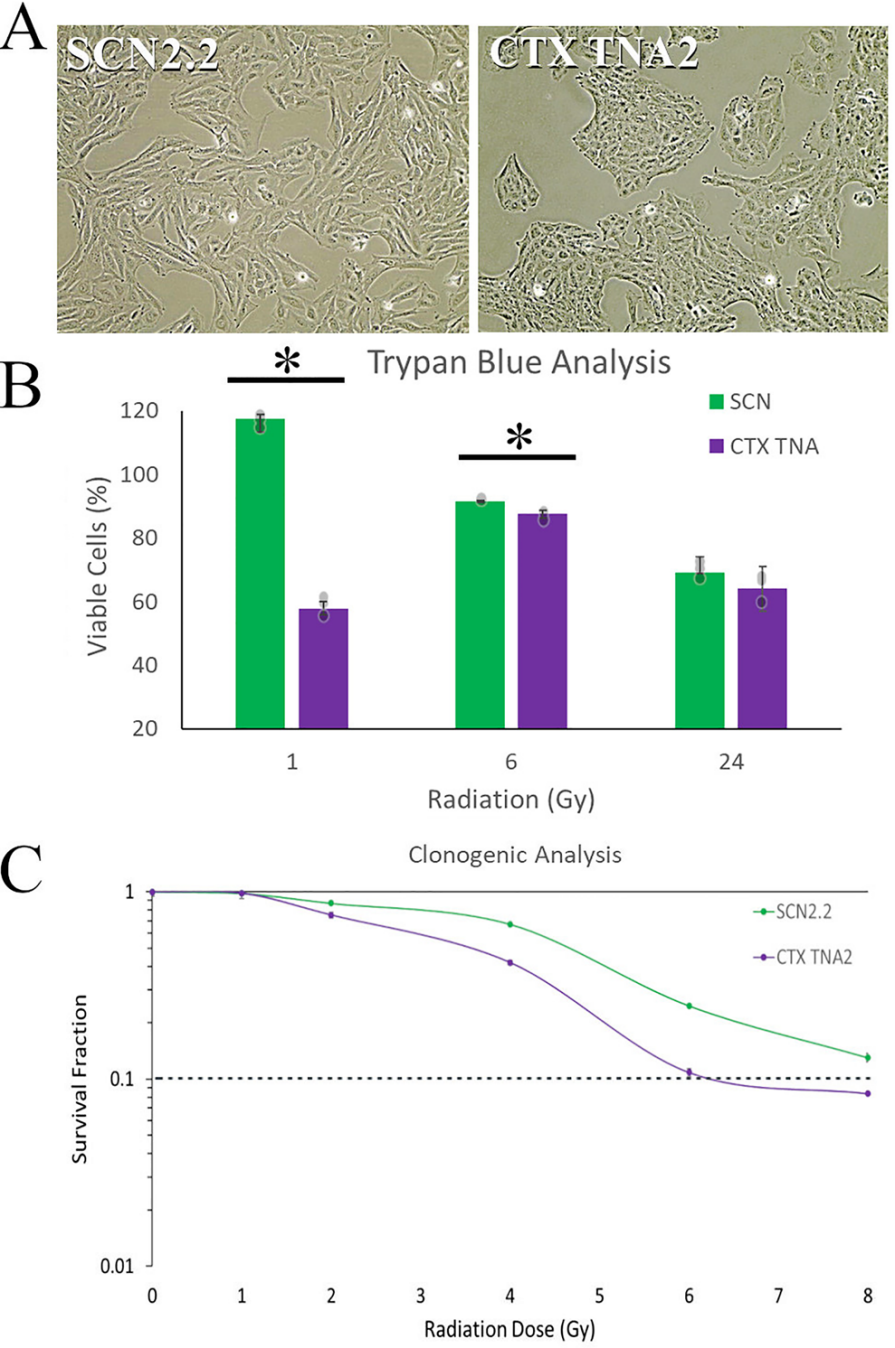

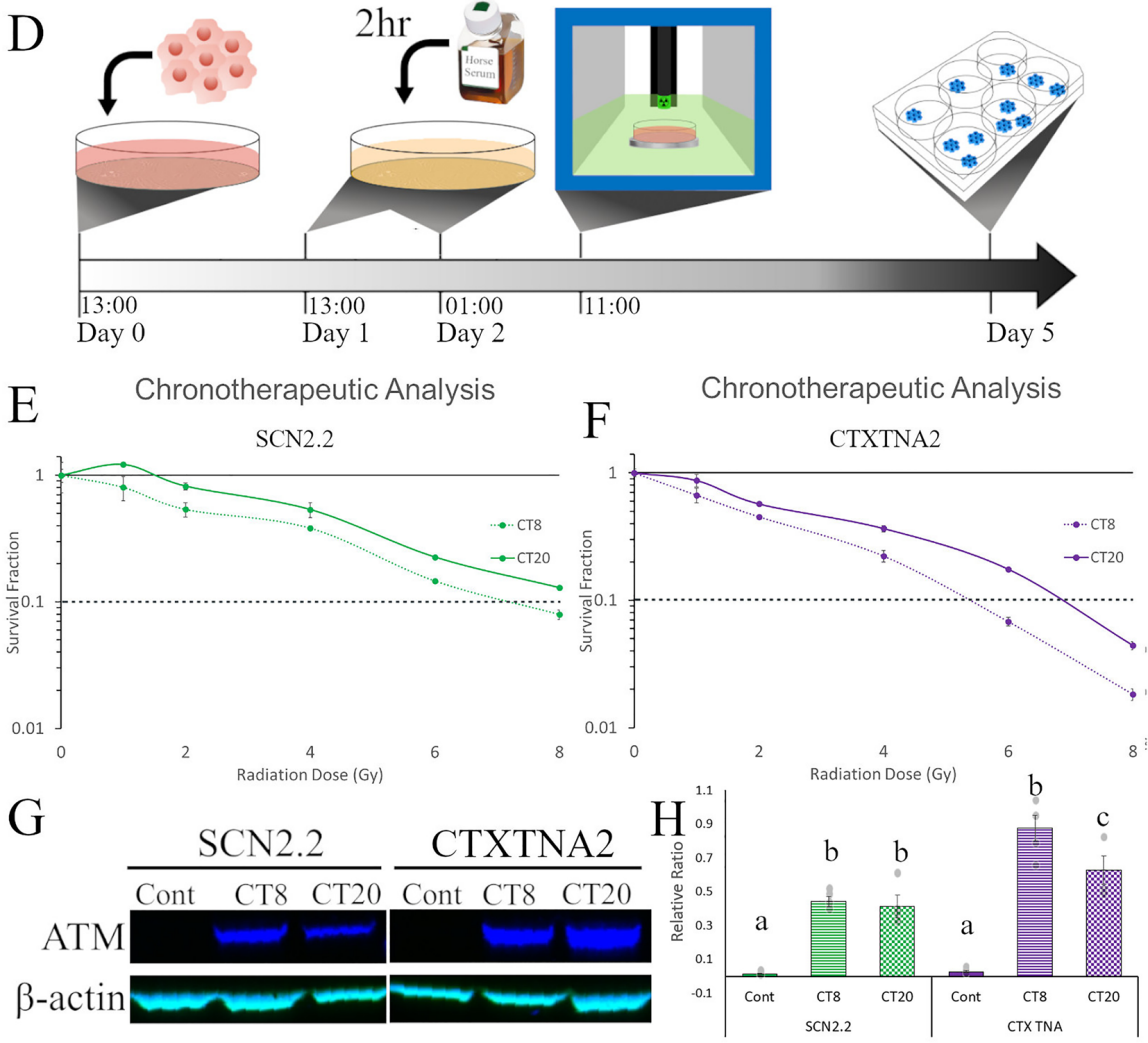
In vitro radiosensitivity of astrocytic cell lines based on region of isolation and treatment timing. (**A**) Photomicrographs of SCN2.2 and CTXTNA2 cells, two astrocytic cell lines with similar morphology. **B.** Quantification of trypan blue postitive cells to determine cell viability 1, 6 and 24 h after 8 Gy radiation, there were significant main effects for time (F(1,18) = 96.834, *p* < 0.001), cell type (F(1,18) = 235.937, *p* < 0.001), and an interaction between time and cell type (F(2,17) = 151.927, < 0.001). Posthoc tests (tukey) were significant if *p* < 0.05. SCN2.2 cells (green bars) survived at significantly higher level 1 (*p* < 0.001) and 6 h (*p* = 0.024) after radiation when compared to the cortical astrocytes (purple bars). (**C**) Clonogenic assay to quantify survival fractions from 0 to 8 Gy in SCN and cortical cells. Again SCN2.2 cells (green line) had better overall survival after radiation when compared to CTXTNA2 cells (purple line). There were significant main effects of both cell line (F(1,24) = 33.228, *p* < 0.001) and radiation dose (F(5,24) = 402.558, *p* < 0.001), and an interaction between cell line and dose (F(5,24) = 6.172, *p* = 0.001). The dotted black line across the graph shows the dose modifying fractors (DMF_10_) levels for both cells lines which was also significantly different between the two cell lines. (**D**) The chronotherapeutic experimental timeline, 1.0 × 10^6^ cells were plated on day 0. Cells were then serum shocked with a 50% horse serum solution for 2 h at circadian time(CT) 8 (01:00, Day 2) or 20 (13:00, Day 1) on the subsequent days. All cells were irradiated on Day 2 at 11:00 and then colonies stained and counted after Day 5. (**E**) Clonogenic assay of SCN2.2 cells irradiated at CT8 (dotted line) and CT20 (solid line). Astrocytes irradiated at CT8 were significantly more sensitive to radiotherapy. There were significant main effects of time (F(1,24) = 7.041, *p* = 0.014) and dose (F(5,24) = 26.995, *p* < 0.001) but no interaction (F(5,24) = 1.081, *p* = 0.396). (**F**) Clonogenic assayof CTXTNA2 cells irradiated at CT8 (dotted line) and CT20 (solid line). Again, astrocytes irradiated at CT8 were significantly more sensitive to radiotherapy. There were significant main effect of time (F(1,24) = 14.099, *p* = 0.001) and dose (F(5,24) = 135.997, *p* < 0.001) but no interaction (F(5,24) = 1.349, *p* = 0.278). (**G**) ATM western analysis of both cell lines when samples were collected at two timepoint post-synchronization. Western blot of ATM (blue bands) and control β-actin (teal bands) in both cell lines show higher presence of ATM after serum shock entrainment (Left panel). A graph of the ATM/β-actin relative ratio (Right panel), shows that CTXTNA cells express more of the protein than SCN2.2 cells. Four replicates of each cell line at the 3 timepoints were compared, there were significant main effects of time (F(2,21) = 83.154, *p* < 0.001) and cell type (F(1,21) = 8.312, *p* = 0.009) and an interaction (F(2,21) = 5.230, *p* = 0.014). Posthoc tests (tukey) were significant if *p* < 0.05. Both cell lines had more protien during CT08 when compared to CT20 but only CTXTNA cells had significant differences between the two (*p* = 0.007). Significance was defined as *p* < 0.05 and indicated by * or a, b, and c with different letters indicating significant differences.

To further examine the variation of radiosensitivity in different brain regions, we conducted *in vitro* experiments in established immortalized rodent cell lines from the SCN (SCN2.2)^48,49^ or Cortex (CTXTNA2)^50^. Both cell lines were originally isolated from fetal or postnatal day 1 rats and display similar morphological features in culture (Fig. 4A) which are characteristic of astrocytes^51^. *In vivo*, the SCN showed lower γH2AX staining during the initial post-radiation sublethal damage repair compared to the cortex, suggesting that cells from this region could be more resistant to irradiation. To determine the gross cell death between cell lines immediately post-radiation (8 Gy), we used trypan blue to stain and then counted cells with a cell viability analyzer after 1 h, 6 h, and 24 h post-irradiation. A two-way ANOVA was significant for time (F(1,18) = 96.834, *p* < 0.001), cell type (F(1,18) = 235.937, *p* < 0.001), and an interaction between time and cell type (F(2,17) = 151.927, <0.001). CTXTNA2 cells had significantly lower percent viability than SCN2.2 cells (Fig. 4B) at 1 h (*p* < 0.001) and 6 h (*p* = 0.024) post-irradiation. Using clonogenic assays, the gold standard for quantifying survival^52^, we further compared the cell lines over a wide range of radiation doses (0–8 Gy). Again, we observed a greater sensitivity to radiation in the cells from the cortex over the SCN (Fig. 4C), with dose modifying factors (DMF_10_) of 1.375 for CTXTNA2 cells as compared to SCN2.2 cells, indicating increased radiosensitivity of CTXTNA2. There were significant main effects of both cell line (F(1,24) = 33.228, *p* < 0.001) and radiation dose (F(5,24) = 402.558, *p* <0.001), and an interaction between cell line and dose (F(5,24) = 6.172, *p *= 0.001). The difference between the cell lines is significant after 2 Gy (t = 3.544, *p *= 0.024) and continue until 8 Gy (t = 6.380, *p* = 0.003). Both trypan blue staining and clonogenic assays show that SCN2.2 cells are more resilient to radiation than the CTXTNA2 cells, which aligns with our γH2AX results.

### Radiation chronotherapy effect impacts healthy tissue in a time-dependent manner

Timing of treatment, chronotherapy, may play an important role in preventing the development of negative side effects caused by radiation^53^. Glioma and fibroblast cells are more sensitive to radiation at circadian time (CT) 20 than those exposed to radiation at CT8^54^. To assess whether the time of treatment played a role in influencing radiosensitivity, we performed clonogenic survival assays in both the SCN2.2 and CTXTNA2 astrocytes at two points in circadian time, CT8 and CT20. To synchronize cells in culture, we used the serum shock method^55^ where plated cells are exposed to high concentration horse serum for 2 h (Fig. 4D) before being irradiated 8 or 20 h post-shock. SCN2.2 cells showed a significant main effect of time (F(1,24) = 7.041, *p* = 0.014) and dose (F(5,24) = 26.995, *p* < 0.001) but no interaction (F(5,24) = 1.081, *p* = 0.396). This indicated that cells were more sensitive to treatment when irradiation was administered (1) during CT8 or (2) as doses increased (Fig. 4E). The DMFs for SCN2.2 cells support the statistical comparison, with the survival of 10% of cells for CT8 at 7.2 Gy and for CT20 at an estimated 8.9 Gy. The CTXTNA2 cells also showed a similar pattern as the SCN2.2 cells (Fig. 4F), with a lower DMF_10_ of 1.26, indicating increased radiosensitivity of CTXTNA2 cells at CT8 compared to CT20. CTXTNA2 cells also showed a significant main effect of time (F(1,24) = 14.099, *p* = 0.001) and dose (F(5,24) = 135.997, *p* < 0.001) but no interaction (F(5,24) = 1.349, *p* = 0.278). Clearly, timing of treatment impacts the sensitivity of these cells but does so in the same direction, greater sensitivity at CT8. These findings are opposite to those we observe in tumor cells and fibroblast^54^.

To examine the underlying differences between cell lines and circadian time in the DNA repair mechanisms critical for double-stranded breaks caused by irradiation, we collected protein samples and ran Western blot analyses for the kinase, ataxia telangiectasia mutated (ATM). The level of ATM activation that occurs in the cell is crucial for DNA damage triggered apoptosis^56^. Four replicates of each cell line at the 3 timepoints were compared, there were significant main effects of time (F(2,21) = 83.154, *p* < 0.001) and cell type (F(1,21) = 8.312, *p* = 0.009) and an interaction (F(2,21) = 5.230, *p* = 0.014). In our samples, we observed higher levels of ATM in both the SCN2.2 and CTXTNA2 cells after the serum shock method when compared to controls (Fig. 4G, H and Supplemental Figure S6). Within the serum shocked samples, CT20 had a lower relative ratio between ATM and control β-actin than CT8 for both cell lines. Additionally, SCN2.2 cells had a lower relative ratio of ATM than CTXTNA2 cells. Higher levels of ATM, in our hands, were associated with lower survival both in cell lines and timing of radiation. This is consistent with prior reports, which showed that genetically modified mice with ATM knocked out are more resistant to radiation-induced DNA damage and apoptosis^57,58^.

### Cranial irradiation impacts the longer-term neuroanatomic structures of the brain

**Figure 5:**
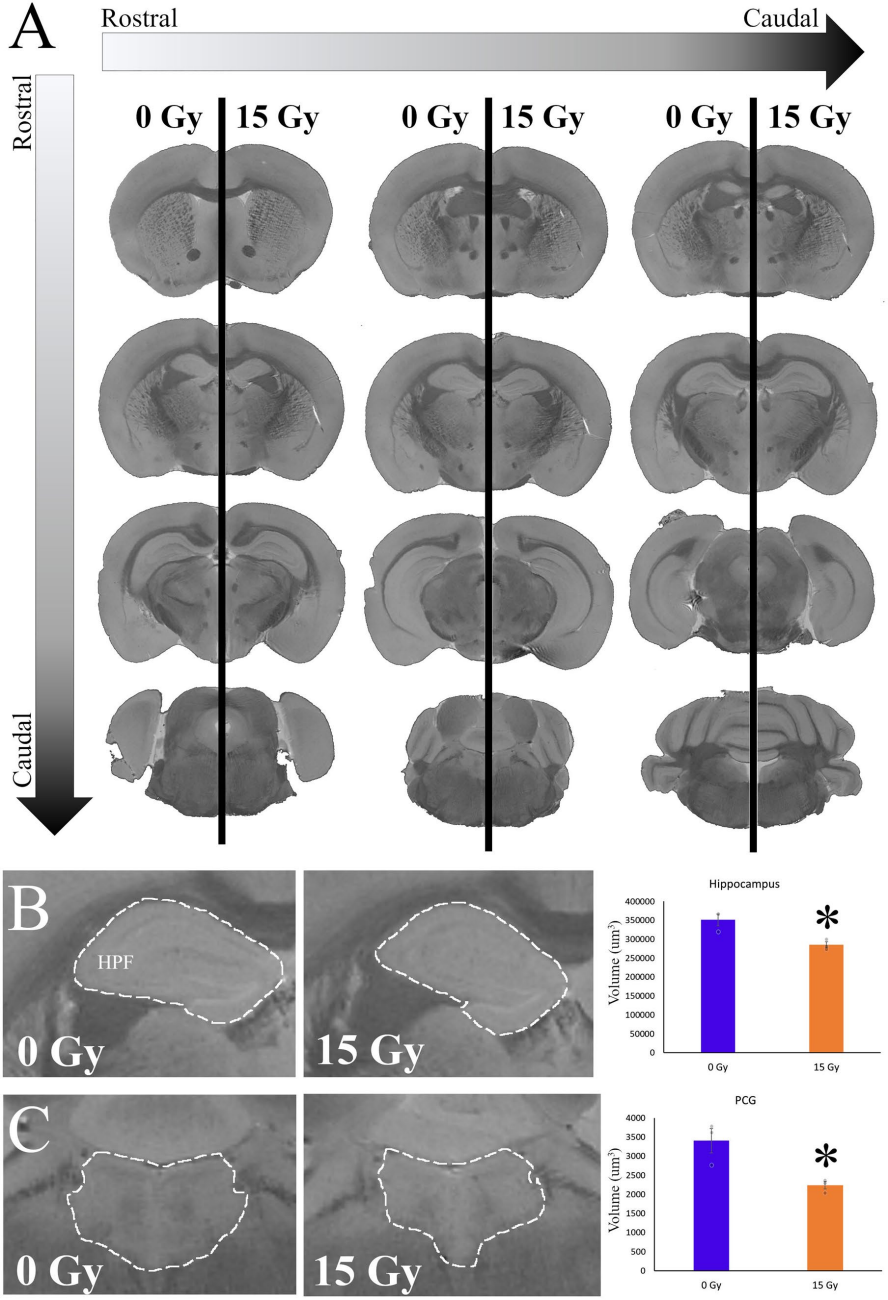
Quantification of structural changes in mice two months post-radiation using high resolution T1-weighted MRI. (**A**) Full rostral to caudal mapping of T_1_ weighted MRI at a resolution of 32 μm^3^ in coronal sections. 2 months post-radiation (15 Gy). Mice were sacrificed 2 months after cranial radiation (n = 3/group), and high resoultion MRI were used to quanitfy volumetric analysis. Sections were organized left to right and top to bottom, in a rostral to caudal manner. On the left are the brain of a representative 0 Gy mouse, while the right portion of the section is a representative 15 Gy mouse. (**B**) Demonstrates the impact of 15 Gy radiation on the volume of the hippocampal formation (HPF). Mice exposed to a control 0 Gy radiation (Left) had larger HPF than those given 15 Gy treatment (Right). These effects were significantly different between the 0 Gy (blue) and 15 Gy (orange) groups (t(4) = 3.833, *p* = 0.019). (**C**) Demonstrates the impact of 15 Gy radiation on the volume of the pontine central grey (PCG). Mice exposed to a control 0 Gy radiation (Left) had larger HPF than those given 15 Gy treatment (Right). These effects were significantly different between the 0 Gy (blue) and 15 Gy (orange) groups (t(4) = 3.504, *p* = 0.025). Significance was defined as *p* < 0.05 and indicated by *.

To understand the longer-term impacts of radiation on brain structures important for regulating sleep, circadian rhythms, and cognition, we irradiated a small group of mice (n = 3/group) using a therapeutic dose and examined MR images of ex vivo brains 2 months post-treatment. Two different pulse sequences were used to either (1) generate high-resolution structural images or (2) T1 maps across the brain. Previous publications in the patient population have shown clear volumetric changes in the hippocampus post-treatment^21^, however, other structures in the cognitive circuit or sleep pathways have not been examined. Here, we quantify volume changes in these structures with our mouse brain using structural MRI, as 3-dimensional fine details of the neuroanatomy are visible with 32µm resolution (Fig. 5A). Volumes of the whole brain (J.D.M) and substructures (D.S.M) were quantified by blinded researchers using Image J software; substructures include Sleep (periaqueductal grey—PAG, and pontine central—PCG), Circadian Rhythms (lateral geniculate nucleus- LGN, habenula—Hb, optic tract- opt), and Cognition regions (hippocampal formation—HPF and cortex -CTX). We observed significant decreases in the volume of regions associated with sleep and cognition but not circadian rhythms (Supplementary Table S2). The most dramatic changes were observed in the hippocampus (t(4) = 3.833, *p* = 0.019; Fig. 5B) and pontine central grey region that encompasses the locus coeruleus (t(4) = 3.504, *p* = 0.025; Fig. 5C).

**Figure 6:**
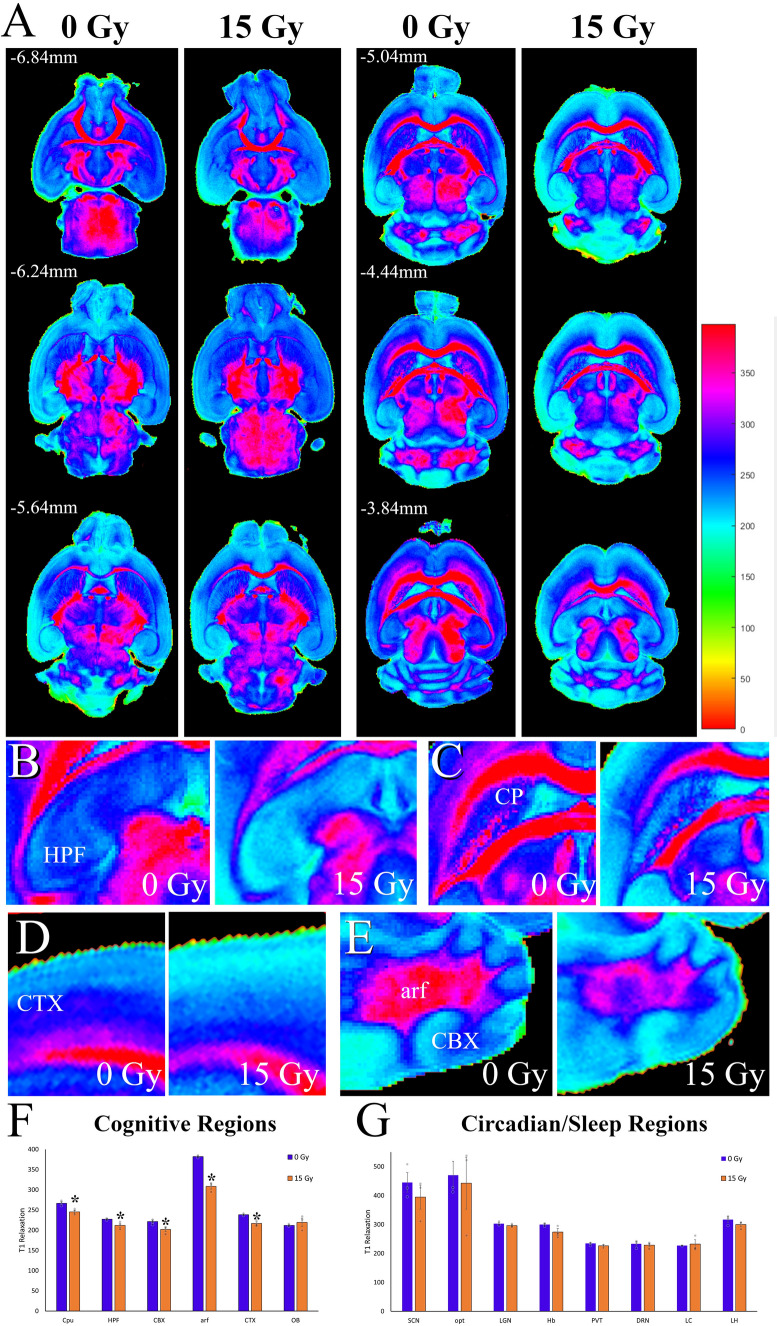
T1-Mapping in mice two months post-radiation. (**A**) Full dorsal to ventral mapping of T_1_ relaxation in horizontal section "Results" months post-radiation (15 Gy). T_1_ relaxation times were calculated using a custom MATLB code and ranged between 0 and 400 ms. The two columns each have both radiation levels, 0 (Left) or 15 (Right) Gy (n = 3/group). Rows represent the dorsal to ventral sections, with the DV coordinates in the top right corner. (**B**) Representative sections of the Hippocampal formation (HPF) with greater relaxation times in the 0 Gy (Left) mouse as compared to the 15 Gy (Right). (**C**) Representative sections of the caudoputamen (CP) with greater relaxation times in the 0 Gy (Left) mouse as compared to the 15 Gy (Right). (**D**) Representative sections of the cortex (CTX) with greater relaxation times in the 0 Gy (Left) mouse as compared to the 15 Gy (Right). (**E**) Representative sections of the cerebellum grey matter (CBX) and white matter (arf) with greater relaxation times in the 0 Gy (Left) mouse as compared to the 15 Gy (Right). The most predominate differences in relaxation time are observed in the white matter portions. (**F**) Graphical comparisons of relaxation time in the cognitve regions of the brain between the two radiation groups, 0 (blue bars) or 15 (orange bars) Gy. All the regions quantified were significant (Cpu, t(4) = 3.724, *p* = −.020; HPF, t(4) = 2.800, *p* = −.049; CBX, t(4) = 2.916, *p* = −.043; arf, t(4) = 10.811, *p* < 0.001; CTX, t(4) = 4.693, *p* = −.009)with the exception of the olfactory bulb (OB, t(4) = 0.050, *p* = 0.525). (**G**) Graphical comparisons of relaxation time in Sleep and circadian regions of interest. None of the areas compared were significantly different between the sham and irradiated groups, although several showed lower non-significant values in 15 Gy mice. Significance was defined as *p* < 0.05 and indicated by *.

Additionally, we used MR imaging with T1-mapping to further interrogate the structural changes occurring within the brains of mice post-radiation. The quantification of longitudinal relaxation time (T1) has been employed to understand disease progression and myelin integrity in neurodegenerative diseases^59,60^. In multiple sclerosis, decreases in T1 relaxation time are associated with edema, inflammation, gliosis, and axonal loss^61^. Here it also provides us with information about regions not clearly visible using high-resolution MRI, such as the SCN. Again, we compared relaxation times between sham and irradiated mice (Fig. 6A) in sleep (DRN, LC, and LH), Circadian rhythms (SCN, LGN, Hb, and opt), and cognitive regions (HFP, CTX, OB, CPu, CBX and arf). Cognitive regions were the only substructures that showed significant decreases in relaxation times (Fig. 6F, G), including the hippocampus (t(4) = 2.800, *p* = −.049; Fig. 6B), caudate (t(4) = 3.724, *p* = −.020; Fig. 6C), cortex (t(4) = 4.693, *p* = −.009; Fig. 6D), and cerebellum (t(4) = 2.916, *p* = −.043; Fig. 6E). These changes in both brain volume and T1 relaxation demonstrate the damage caused by radiation, supporting the concept that this injury may impact cognitive function and worsen sleep symptoms.

## Discussion

With improvements to cancer treatment leading to better survival, treatment-related toxicities and symptoms are increasingly important. However, understanding the mechanisms that lead to the development of these toxicities and symptoms is difficult in human subjects. Here, we demonstrate a consistent and reproducible mouse model of cranial radiation-induced hypersomnolence that recapitulated the symptoms experienced by patients with primary brain tumors who underwent radiotherapy. In mice, irradiation negatively impacted both sleep-like behavior and general activity in a dose-dependent manner (Fig. 1) and was sustained over a month (Fig. 2). We further proposed that radiation differentially damages brain regions important for driving the development of common symptoms. Specifically, having a greater negative impact on sleep and cognitive circuits. Immediate DNA damage caused by radiation showed higher levels of the γH2AX marker 1 h post-radiation in the cognitive and homeostatic sleep pathways, including the lateral hypothalamus, dorsal raphe, and locus coeruleus, but not the circadian suprachiasmatic nucleus (Fig. 3). At the cellular level, we continued to observe the region-specific effect of radiation. Astrocyte cell lines isolated from the SCN were less sensitive to irradiation than those from the cortex as measured by trypan blue and clonogenic assays (Fig 4A–C). When we examine the longer-term impact of radiation on the brain using MRI, we found that both sleep and cognitive regions were negatively affected by the treatment with both having smaller volumes (Fig. 5). Cognitive regions also, had further alterations in T_1_ relaxation post-radiation (Fig. 6). Interestingly, the circadian system, including the non-image forming visual pathway, again remained unaffected in the longer term using these measurements. Cranial irradiation impacts the behavioral expression of activity and sleep and directly damages regions of the brain that drive these behaviors in mice, suggesting a possible mechanism for the development of symptoms commonly experienced by patients.

Mice exposed to high levels of cranial radiation had distinct behavioral changes that were like symptoms observed in PBT patients, including the suppression of daily activity and heightened levels of sleep during the active phase. Oncologic therapies can limit the normal daily activity of patients, by reducing stamina and causing fatigue^62,63^. In primary brain tumor patients, levels of physical activity are associated with patient-reported fatigue^64^, physical function^65,66^, and survival^67^. Further analysis of physical activity levels using smart wearable devices are being explored in two current clinical trials (NCT04669574 & NCT02781792), including the analysis of circadian rhythms and physiological sleep which have not yet been explored in PBT patients. Sleep disturbances, specifically excessive daytime hypersomnolence, is one of the most common symptoms in primary brain tumor patients^3,4^. Direct links between these heightened sleep issues and other negative symptoms, such as depression, fatigue, and cognitive impairments, have been demonstrated in the PBT population^67–69^. Unfortunately, these co-morbidities make it hard to disentangle the impacts of sleep disturbance on survival^70,71^. In other cancers^72,73^ and the healthy population^74,75^, disruption of sleep has been shown to have a negative relationship with survival outcomes. Our mouse model of C-RIH mice allows for the testing of these relationships; we predict that the introduction of tumors in irradiated mice would lead to faster disease trajectory and the further development of other behavioral complications which may be further exacerbated in carriers of specific clock gene polymorphisms^29^.

Another factor that could impact the susceptibility of patients to developing SD is age. Malignant primary brain tumors occur at the highest frequency in people greater than 60 years of age^76^. Age greatly impacts the expression of both homeostatic and circadian sleep control mechanisms^32^, therefore, age-related dysregulation of these neurological networks magnifies the effects of further SD on normal functions, like cognition^77^. In the current study, only young (6 week old) mice were used so using older mice for further experimentation into the relationship between C-RIH and age may provide additional insight into the pathogenesis of radiation-induced SD. Overall, our mouse model demonstrates a behaviorally accurate tool by which to explore several questions about the physiological and neuroanatomic changes that promote the development of hypersomnolence.

Studies of neurocognitive decline with radiotherapy have focused on the hippocampus^17–21^, demonstrating that sparing hippocampal radiation reduces the impact on cognitive function^22–24^. Our works demonstrated that there are many regions associated with cognition that are impacted by radiation with only the olfactory bulbs having unaffected T1 relaxation after treatment exposure. The olfactory bulb appeared to be spared and interestingly, was recently shown to be a radioresistant niche for tumor cells^78^ and is home to the largest number of neural progenitor cells in the mouse brain. In our study, brain regions that drive sleep in the homeostatic pathways also had high levels of DNA damage staining, including the lateral hypothalamus, dorsal raphe, and locus coeruleus. Importantly, decreases in cells important for regulating sleep in these regions have been associated with the development of SD in other diseases^79–81^. Hypersomnolence, specifically, is observed in rodents with lesions in the dorsal raphe^82,83^ or loss of critical neurons in the lateral hypothalamus^84,85^. MRIs performed on animals at later time points showed possible loss of cells within these regions as significant decreases in volume were observed in the pontine central gray, which included the locus coeruleus, and a trend toward significance in the periaqueductal grey, which included the dorsal raphe, but without apparent alteration in T1 relaxation for any sleep-regions assessed. The short- and longer-term impact of radiotherapy on sleep and cognition regions of the brain are clear in our model and suggests a possible mechanism for the development of symptoms in patients.

Circadian rhythms in the animal-based experiments, both behavioral and neuroanatomical, do not demonstrate significant effects like those observed for homeostatic sleep. These findings were surprising as our cell culture experiments showed distinct circadian time-based differences in radiosensitivity in astrocytes and other publications have shown these effects in glioma tumor cells^55,86,87^. Interestingly, the expression of circadian rhythms in clock genes and the clock-controlled genes, which they modulate, varies in different brain areas with the phases sometimes not matching the SCN^88–90^. This suggests the possibility of time-based differences in radiosensitivity across different regions. In fact, in a publication by Mure et al. the expression of the kinase critical for DNA damage triggered apoptosis, ATM, was examined in different regions of the primate brain (supplemental material) and appeared to have varying amplitudes, periods, and phase from the SCN^90^. The lack of significant effects in the SCN or other circadian visual areas could be linked to a limitation in our experimental design; all animals here were given radiation only at one time of day during the inactive phase, daytime. Our systematic review of the use of radiation chronotherapy in the oncology patient population^53^ suggests that timing of treatment impacts symptoms with worst outcomes when treatment was given during the afternoon. We might, therefore, see greater symptoms if animals were treated during their late active phase. This study also had a relatively small size for the MRI analysis and was only conducted in male mice, two factors that could impact the ability to generalize significance. These studies support the need to further investigate the timing of radiotherapy in our mouse model and the PBT population.

In conclusion, our findings show that cranial irradiation in mice can induce symptoms that recapitulate the human experience. Mice displayed decreased general activity and increased daytime sleep in a dose-dependent and sustained manner. In C-RIH mice, damage to the brain is characterized through γH2AX staining and MRI suggests that homeostatic sleep and cognitive regions are more responsive to ionizing radiation. Further, region-specific effects of radiation were also supported by *in vitro* experiments, which demonstrated higher sensitivity in cortical cells as compared to those from the SCN. Further exploration of the functional changes to these regions is required to understand if the damage and atrophy observed here impacts behavior directly. The successful development of this model will hopefully enable the testing of alternative treatment schedules or preventative therapies that will reduce the incidence and severity of these devastating symptoms while maintaining the anti-cancer activity of the radiation treatment.

## Methods

### Animals and housing

Thirty-two adult, male C57BL/6 mice were obtained from Charles River Laboratories (CRL, Dublin, VA). Animals were singly housed in PhenoTyper 3000 cages (Noldus Information Technologies; Wageningen, Netherlands) with infrared sensitive camera with three arrays of infrared LED lights and monitored continuously using the Ethovision XT 14 Software (Noldus Information Technologies) or housed in standard mouse cages. Food and water were provided ad libitum and lights were on a 12:12 Light:Dark (LD) schedule with intensities ranging from 0 to 60 lum/ft2. All experimental procedures were approved by the Institutional Animal Care and Use Committee at the National Cancer Institute and followed the National Institute of Health Guide for the Care and Use of Laboratory Animals (NIH Publications No. 80-23) revised 1996.

### Experimental design

Experiments were designed in compliance with the ARRIVE guidelines (https://arriveguidelines.org). Animals were used to establish a behavioral model of radiation-induced hypersomnolence and to then quantify the neuroanatomical changes that occur post-irradiation. All mice were irradiated using a small animal Pentax x-ray irradiator machine. Prior to irradiation, animals were injected intraperitoneally with 0.1–0.5cc of anesthesia (ketamine: 80–120 mg/kg and xylazine: 5–25 mg/kg) for immobilization. Once anesthetized, mice were transferred into a plexiglass pie holder with a custom lead shielding lid. To isolate radiation to only the brain and ensure that the eyes, nose, and body are spared, the custom lead shield was designed with small holes in the shielding where only the skull is exposed to irradiation. Post-irradiation, mice were monitored for up to 2 h on a heating pad until mice regain consciousness then transferred into their home cages.

Two behavioral experiments were conducted to (1) establish a dose response curve and identify the optimal radiation dose for inducing hypersomnolence and (2) track the behavior of mice at the optimal dose across one month (longer-term monitoring). Dose response mice were exposed to one of five doses of radiation, 0 Gy, 2 Gy, 5 Gy, 10 Gy and 15 Gy. Effects in these groups were compared between low radiation, defined as 0 Gy and 2 Gy, and high radiation doses, defined as 5 Gy, 10 Gy and 15 Gy. The longer-term monitoring animals in the second behavioral experiment were irradiated with sham (0Gy) or 15 Gy. To examine general activity and sleep patterns for the two experiments, behaviors were recorded in the PhenoTyper 3000 cages. All experiments had 10 days of baseline data recording prior to irradiation, with only 7 days used to compare to post-radiation behaviors. General activity between the cohorts for the dose response experiment were monitored for 10 days post-irradiation and for the 20 days in the longer-term monitoring experiment. Total distance traveled and velocity were examined across twenty-four hours at the hour and day/night levels using the standard Ethovision software. Sleep-like behavior, eating, grooming, and drinking for animals were also monitored using an add-on Mouse Behavioral Module. Further variables of sleep-like behavior, number of sleep bouts and total bout duration, were calculated by summing all the one-minute bins that were greater than 50s and occurred concurrently. Circadian parameters were measured using methods established in Shuboni-Mulligan et al.^32^, we plotted distance travelled in 10min interval and quantified amplitude, activity onset, offset and precision for the final 3 days (days 8–10) of analysis and compared it to the initial 3 baseline days.

Two neuroanatomical experiments were conducted to examine (1) the short-term effects of DNA damage across the whole brain using histological techniques and (2) the longer-term impact of radiation on brain volume and T1 mapping in regions-of-interest associated with sleep, circadian rhythms and cognition using neuroimaging. All animals were irradiated at 15 Gy and tissues were collected at 1 h post-irradiation for the short-term experiment and after 22 days for the longer-term experiment. To collect brains for histology and MRI, animals were anesthetized using isoflurane and transcardially perfused using 4% paraformaldehyde. Brains were removed from the skull and postfixed for 2 h before being stored in 0.1M phosphate buffer saline (PBS) solution.

### Ex vivo whole brain magnetic resonance imaging

To compare the longer-term impact of radiation on brain structures, whole brains were extracted from euthanized mice. Whole brains were kept in PBS to prevent dehydration until they were imaged using a 14.1T/4cm Bruker microimaging scanner (Bruker Corporation, MA). Before scanning, brains were placed in a tube filled with Flourinert (3M Company, MN). T1 maps were acquired using a RAREVTR sequence with the following parameters: TE = 12.557ms, TR = 35 50 80 120 160 200 400 800 1200 2000 4000ms, FOV=1.72 × 1.28 × 0.96 cm, resolution = 32 × 32 × 8 µm, and total scan time = 15 h 26 m. For volumetric analysis, brains were placed into gadolinium (0.1M gadopentetate dimeglumine, Magnevist) doped PBS for 48 h and then imaged for high resolution 3D images with a FLASH sequence using the following parameters: TE = 5ms, TR = 50ms, FOV =1.6 × 0.92 × 1.28 cm, resolution = 32 × 32 × 32 µm, Averages = 16 and total scan time = 20h 28 m. Brain volumes were quantified by blinded researchers (D.S.M. and J.D.M.) as previously described^32^.

### Histological and immunohistochemical Techniques

Whole brains were transferred into 30% sucrose solution for a minimum of 48 h to ensure cryoprotection during sectioning. Brains were blocked by removing the olfactory bulbs and brain stem caudal to the cerebellum. Using Optimal Cutting Temperature solution (Tissue-Tek OCT, Sukura Fineteck USA, CA), brains were attached to specimen disc. Once the base of the brain was attached, 30% sucrose solution was coated on the surface of the specimen and then was rapidly frozen using powered dry ice. Specimen discs were placed into a Leica CM1860 for 1 h prior to sectioning to allow the brain to come to chamber temperature (−19 °C), which ensures even slices. The entire length of the brain from anterior commissure to the cerebellum was section at 30 µm and grouped into three replicates for complimentary staining, one series was used for and another γH2AX for DAPI.

Sham and Irradiated brains sections were stained for either γH2AX or counterstained for DAPI. To stain for γH2AX, free floating sections were first washed 6 times in PBS and then blocked**-**in normal goat serum (005-000-121; Jackson ImmunoResearch Laboratories Inc, PA) for one hour. After one 10 min rinse in PBS, sections were transferred into a primary antibody, mouse anti- γH2AX (JBW301; Millipore Sigma, MA), for 72 h at 4°C. Sections were then washed 3 times for 10 min/wash and transferred into secondary antibody, goat anti-mouse (115-000-003; Jackson ImmunoResearch Laboratories Inc, PA), for one hour at room temperature. Finally, sections were incubated in a Vectastain ABC-HRP solutions (PK-4010; Vector Laboratories, CA) for 30 min and then reacted for 45 seconds with DAB peroxidase substrate with nickel (SK-4100; Vector Laboratories, CA) to produce a blue stain. Sections were washed 6 times for 10 min/wash to stop the staining process. Brains were mounted onto slides, dehydrated and coverslipped with PermountTM solution (Fisher Scientific, MA). The number of cell nuclei observed across the brain may play an important factor in that understanding DNA damage levels in different brain regions of interest, we therefore used a complementary replicate to counter stain with DAPI. Unstained brain sections were mounted onto gelatinized slides, allowed to dry**,** and then coverslipped with ProLong Diamond Antifade Mountant with DAPI. Sections were allowed to cure hard for 24 h before imaging.

Light microscopy at 10× magnification was used to examine and compare γH2AX staining in irradiated and sham animals using an Olympus BX43 scope (DSM). One representative irradiated animal had complete sections imaged across the brain. These images were stitched together manually by DSM using GIMP 2.10.8 software. Identical whole section images of the DAPI staining in the same animal were acquired using the fluorescent feature of a Nikon confocal microscope. The NIS-Elements software equipped on the Nikon microscope automatically merged the DAPI images for each section of interest.

### In vitro cell culture techniques

Astrocyte cell lines were acquired from Kerafast, Inc. (Boston, MA) for SCN2.2 cells and American Type Culture Collection (ATCC, MD) for CTX TNA (ATCC CRL-2006) cells. Both cell lines were maintained in Minimal Essential Media with nonessential ammino acids (10370021; Gibco Laboratories, MD) with 10% Fetal Bovine Serum, 4.5mL 45% glucose per 500mL (A2494001; Gibco Laboratories, MD), 1% L-glutamine (25030149; Gibco Laboratories, MD) and 1% Antibiotic-Antimycotic. Cells were irradiated using a 137Cs source (Mark I irradiator; J. L. Shepherd and Associates, San Fernando, CA) at a rate of 1.896 Gy/min. To test survival between the cell lines, we used trypan blue for short-term effects and clonogenic assays for longer-term effect.

Short-term effects of radiation were tested in cells seeded in 6-well plates at 1x10^6^ cells per well, each cell line had 3 replicates per plate. Cells were irradiated at 8 Gy for 4 min and 13 seconds, after 1 h post radiation cells were trypsinized, washed and resuspended in media. Cell survival was quantified using a Vi-CELL XR Cell Viability Analyzer (Beckman Coulter Inc., CA), cells number and viability was determined with trypan blue. Longer-term effects of radiation were tested in cells using clonogenic assays^92^. Cells were seeded on 6-well plates in triplicate at two concentrations per radiation dose and allowed to attach for a minimum of 24 h prior to radiation. Plates were irradiated at 0 Gy (sham), 1 Gy, 2 Gy, 4 Gy, 6 Gy and 8 Gy for both cell lines and cells were allowed to grow for 6 days, until the colonies in the sham had at least 25 cells in each cluster. Cells were stained with 0.5% crystal violet for 10min and washed with water 3 times before drying upside-down for 48 h. The number of colonies counted blind to the group with a stereomicroscope (DSM), and the surviving fractions were calculated and normalized to unirradiated shams, respectively.

Chronotheraputic effects were tested in vitro by synchronizing cells using the serum shock method^51^. In brief, cells were seeded and allowed to attach to 6-well plates, then media was washed with PBS and cells incubated in a 50% horse serum (16050130, Gibco Laboratories, MD) solution for 2 h. Serum shock was conducted at 20 h (CT20) or 8 h (CT8) before radiation was given, each timepoint had a separate collection of clonogenic plates (0 Gy (sham),1 Gy, 2 Gy, 4 Gy, 6 Gy and 8 Gy). Samples of protein were also collected from Control (un-pulsed), CT20 and CT8 for western blot analysis of ATM expression, a kinase recruited and activated by DNA double-strand breaks. Cells for the Western blot were plated on 100mm petri dished at a concentration of 1 × 106 cells per plate and allowed to attach overnight. At CT20 and CT8, the plates were serum shocked on the following day, so that all samples would be collected at the same time. To collect protein samples, cells were washed 2 times with PBS then 0.2 mL of RIPA lysis buffer (K2031-75; USbiological, MA) was added to the dish and scraped to dislodge cells into solution then immediately frozen at −80°C.

Immediately before running, the cell lysates were thawed then centrifuged 2x at 10,000G for 10 min and the supernatant was preserved each time for final testing. Levels of protein were determined with the Qubit 4 Fluorometer (Invitrogen, CA) and assay kit (Q33212; Invitrogen, CA). Sample wells were loaded with 50µg/µL of protein and run on NuPAGE Bis-Tris gels (Thermofisher, MA), then transferred to PVDF membranes (IB24002; Thermofisher, MA) using the iBlot2TM Gel Transfer Device (IB21001; Thermofisher, MA). Membranes were stained using primary antibodies for ATM (1:1000; ab81292, abcam, MA) and β-actin (1:5000; ab8226, abcam, MA) for 24 h at 4°C, followed by 1 h at room temperature in secondary antibodies (1:5000) for IRDye 800CW Goat anti-Mouse (926-32210; Li-Cor Biotechnology, NE) and IRDye 680CW Goat anti-Rabbit (926-68071; Li-Cor Biotechnology, NE). Membranes were then imaged with ChemiDoc Touch Gel Imaging System (Bio-Rad, CA) and analyzed using Image J software (https://imagej.net) to determine relative ratio of protein levels.

### Statistical analysis

All analyses were completed using SPSS statistical software (IBM, Armonk, NY). Comparisons in behavioral experiments were analyzed using mix-model ANOVAs with the days as a within subject variable and the radiation dose as a between subject variable, significant interactions were compared using post hoc tests with Tukey corrections. Paired and independent samples t-tests were used to compare groups of interest for sleep and circadian analysis. GammaH2AX and DAPI levels within areas of interest were compared for staining level between brain regions within the irradiated group; high levels of staining were dark blue, while low staining levels were whiter. Volumetric and T1 differences were compared between the two radiation groups using an independent samples t-test. Finally, comparisons for the cell culture work mix-model ANOVAs with the radiation dose as a within subject variable and cell type or time-of-day as a between subject variables. Independent samples t-tests were used to compare groups of interest within the ANOVA and to compare the dose modifying factor (DMF_10_) between curves. For all experiments, statistical significance was set at *p* < 0.05.

## Supplementary Information

Supplementary Information.
