# Supplementary material for: Histological analysis of sleep and circadian brain circuitry in cranial radiation-induced hypersomnolence (C-RIH) mouse model
Source: Sci Rep. 2022 Jul 1;12:11131. doi: 10.1038/s41598-022-15074-0 (PMC9249744; doi:10.1038/s41598-022-15074-0)
Supplement: Supplementary file 1 — Supplementary Information. [file 41598_2022_15074_MOESM1_ESM.docx]

**Histological analysis of sleep and circadian brain circuitry in cranial radiation-induced hypersomnolence (C-RIH) mouse model**

Dorela D. Shuboni-Mulligan^1*#^, Demarrius Young Jr.^1#^, Julianie De La Cruz Minyety^1^, Nicole Briceno^1^, Orieta Celiku^1^, Amanda King^1^, Jeeva Munasinghe^2^, Herui Wang^1^, Kendra Adegbesan^1^, Mark R. Gilbert^1^, DeeDee Smart^3^, and Terri S. Armstrong^1^

#Co-First Authors

*Corresponding Author

^1^Neuro-Oncology Branch, National Cancer Institute, National Institutes of Health, Bethesda, MD, USA. ^2^Mouse Imaging Facility, National Institute of Neurological Disorder and Stroke, NIH, Bethesda, MD, USA. ^3^Radiation Oncology Branch, Center for Cancer Research, National Cancer Institute, National Institutes of Health, Bethesda, MD, USA.


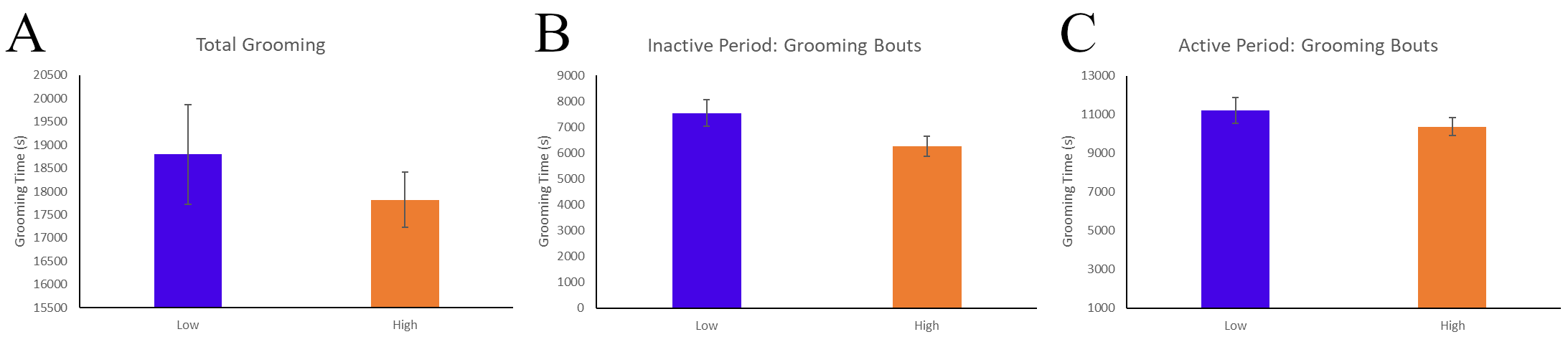


**Supplementary Figure S1. Grooming behavior trending toward significant in dose response experiment.** Low radiation (blue) and high radiation (orange) groups were compared across 24hr (a, Total Grooming), during 12 h of the inactive period (b) or active period (c).

**Supplementary Table S1. Analysis of circadian rhythms variables in the dose response experiment. Using the raw 10-min level data of distance traveled, Cosinor analysis (Roberto Refinetti’s Circadian Cosinor Software, http://www.circadian.org/ main.html) determined the amplitude of the daily rhythms for all animals. To further probe circadian variables for activity, actograms of total distance traveled in 10 min bins were generated for all animals and scored for activity onset and offset by two blinded scorers (DSM and DY). Precision was defined as the variability in the phase angle of entrainment across the days analyzed.**


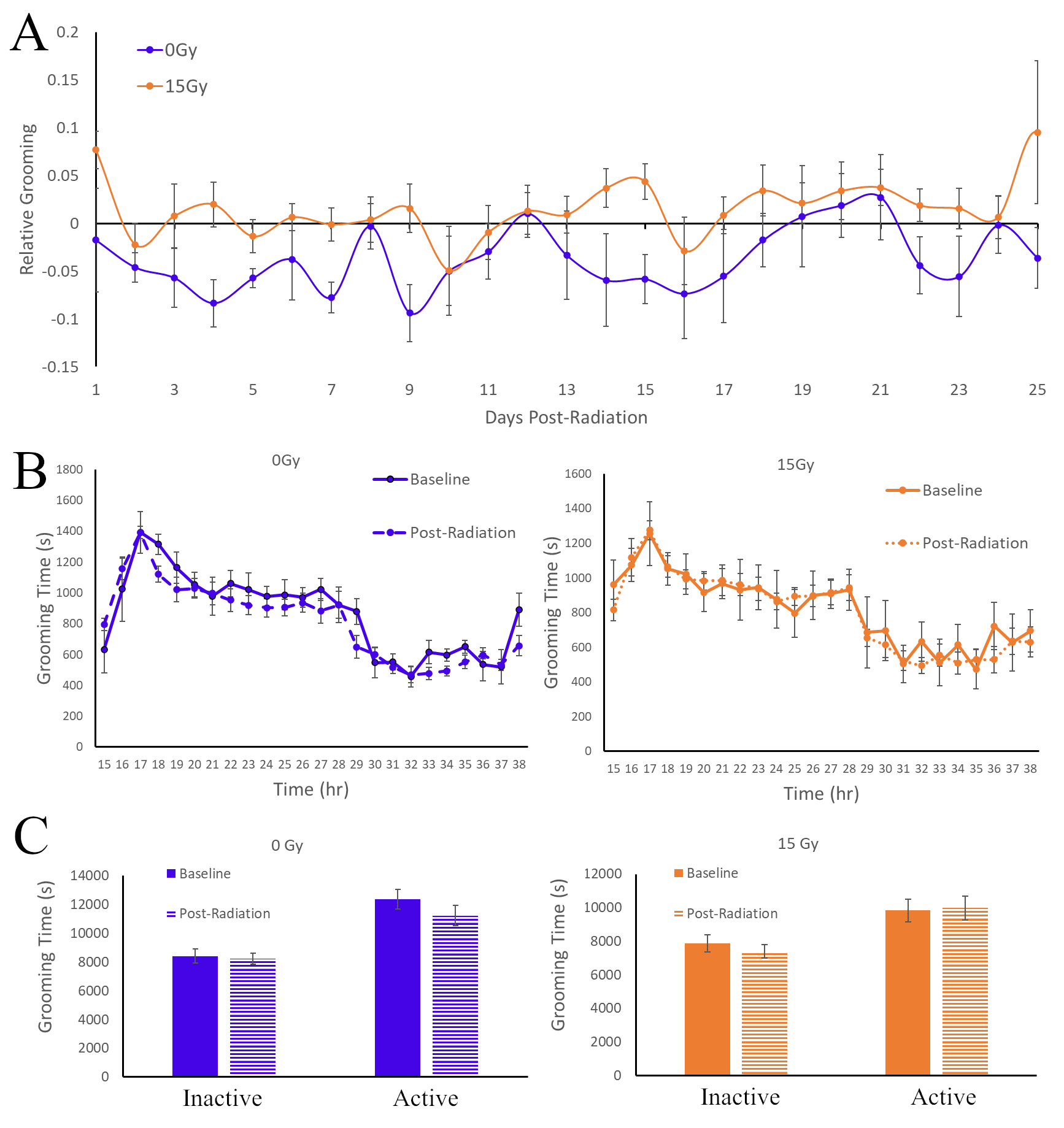


**Supplementary Figure S2. Grooming behavior trending not significant in dose response experiment.** (A) Total daily relative grooming across time, corrected with baseline levels of behavior. Mice receiveing sham irradiation (0 Gy, blue) or 15 Gy (orange) are depicted in all graphs. (B) Daily profiles of mice graphed across 24hrs when average at baseline and after irradaition for the sham (left) and 15 Gy (right). Solid lines indicate the baseline levels of grooming, while dotted lines are for after the treatment is performed. (C) Inactive/Active graphs showing levels of grooming based on time of day. Solid bars indicate the baseline levels of grooming, while stripped bars are for after the treatment is performed.


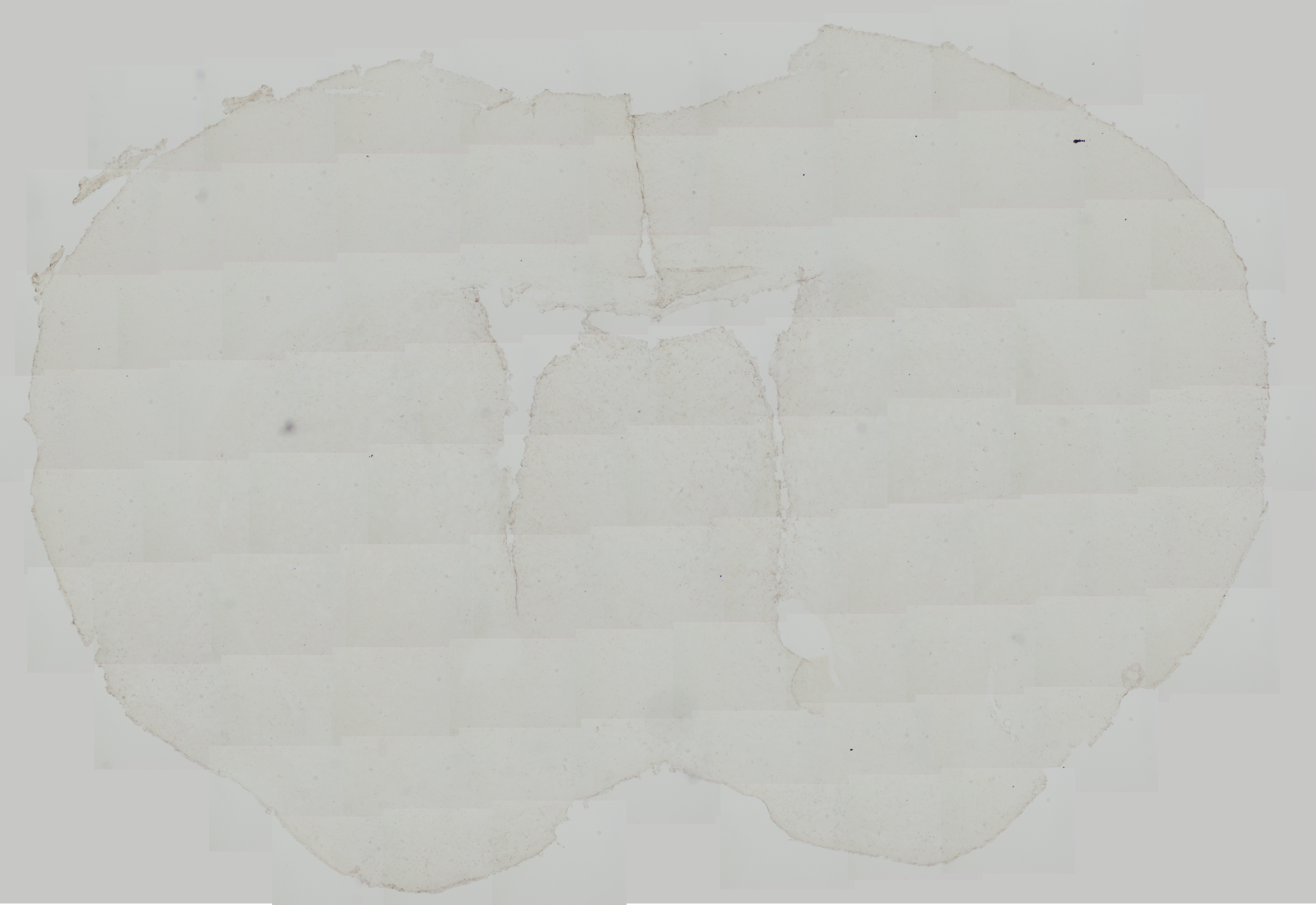


**Supplementary Figure S3. Histological control for γH2AX in animal given sham irradiation.** No background staining is observed in the section as compared to the 15 Gy mice.


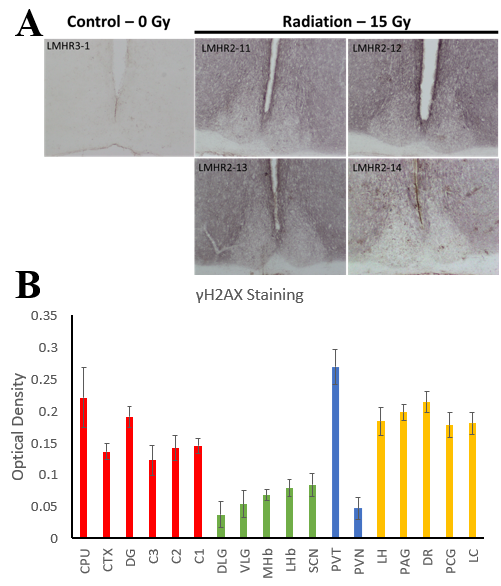


**Supplementary Figure S4. γH2AX staining in the SCN of four irradiated mice when compared to control. (A)** Levels of staining are markedly lower in the SCN than the surrounding hypothalamic regions. (B) The optical densities of regions in across different brain areas, mean ±SEM. Red indicates cognitive areas, Green indicates the Circadian Visual System, Blue indicates the Hormonal Control, and Yellow indicates Sleep areas.


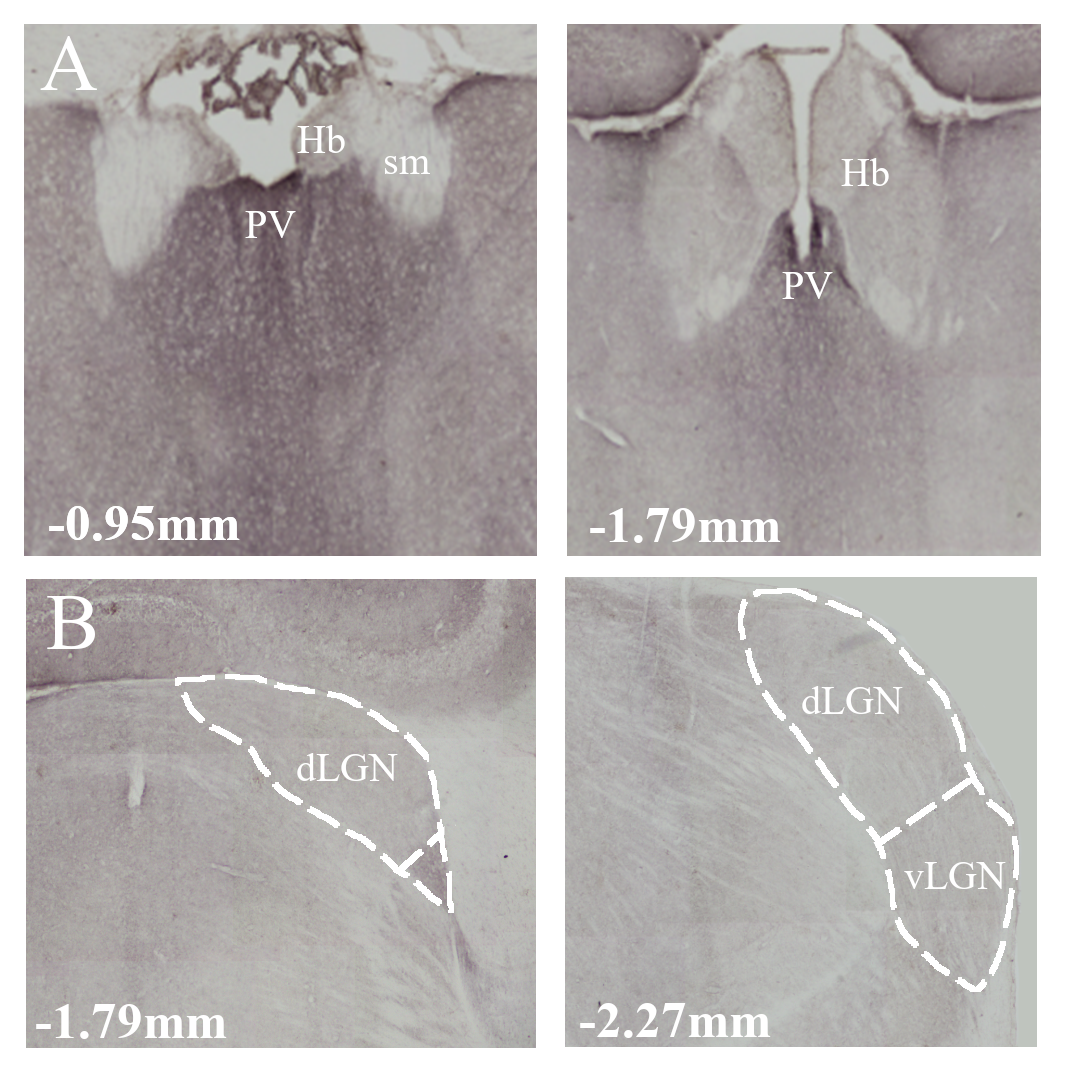


**Supplementary Figure S5. γH2AX staining in the Paraventricular Nucleus of the Thalamus and the Lateral Geniculate Complex of the circadian visual system.** (A) The paraventricular nucleus of the Thalamus (PV) shows higher levels of staining than other thalamic regions. The habenula oberved in these sections also shows lower levels of staining that the PV. (B) The lateral geniculate complex including the dorsal (dLGN) and ventral (vLGN) have the typical lower staining levels observed across the thalamus.

**
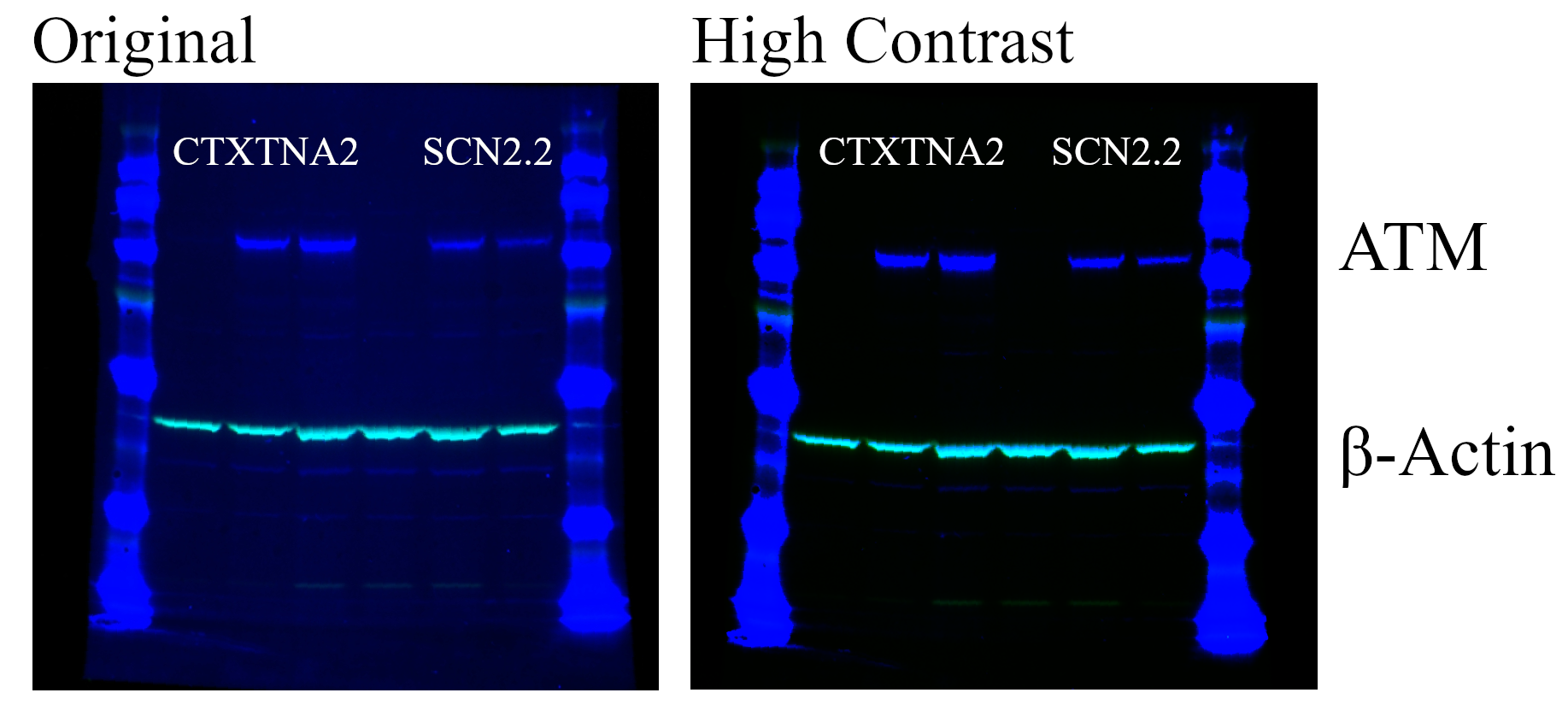
**

**
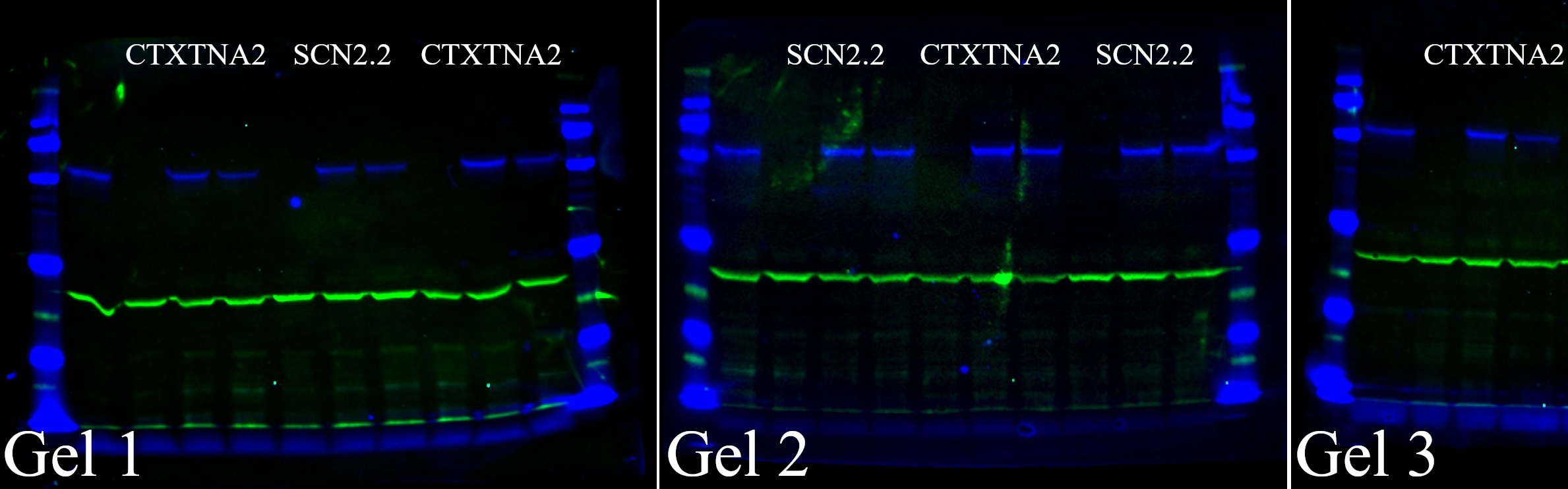
**

**Supplementary Figure S6. Whole ATM western imaged in β-actin (green) and ATM (blue).** Images were taken in a single membrane using two different fluorescent probes.

**Supplementary Table S2. Volumetric Analysis of brain regions important for cognition, sleep and circadian rhythms.**
